# Deprescribing medication in very elderly patients with multimorbidity: the view of Dutch GPs. A qualitative study

**DOI:** 10.1186/1471-2296-13-56

**Published:** 2012-07-09

**Authors:** Jan Schuling, Henkjan Gebben, Leonardus Johannes Gerardus Veehof, Flora Marcia Haaijer-Ruskamp

**Affiliations:** 1 Department General Practice, University Medical Centre Groningen, A.Deusinglaan 1, 9713AV, Groningen, The Netherlands

**Keywords:** General practice, Frail elderly, Polypharmacy, Withdrawing treatment, Preventive therapy, Quality of life

## Abstract

**Background:**

Elderly patients with multimorbidity who are treated according to guidelines use a large number of drugs. This number of drugs increases the risk of adverse drug events (ADEs). Stopping medication may relieve these effects, and thereby improve the patient’s wellbeing. To facilitate management of polypharmacy expert-driven instruments have been developed, sofar with little effect on the patient’s quality of life. Recently, much attention has been paid to shared decision-making in general practice, mainly focusing on patient preferences. This study explores how experienced GPs feel about deprescribing medication in older patients with multimorbidity and to what extent they involve patients in these decisions.

**Methods:**

Focusgroups of GPs were used to develop a conceptual framework for understanding and categorizing the GP’s view on the subject. Audiotapes were transcribed verbatim and studied by the first and second author. They selected independently relevant textfragments. In a next step they labeled these fragments and sorted them. From these labelled and sorted fragments central themes were extracted.

**Results:**

GPs discern symptomatic medication and preventive medication; deprescribing the latter category is seen as more difficult by the GPs due to lack of benefit/risk information for these patients.

Factors influencing GPs’deprescribing were beliefs concerning patients (patients have no problem with polypharmacy; patients may interpret a proposal to stop preventive medication as a sign of having been given up on; and confronting the patient with a discussion of life expectancy vs quality of life is ‘not done’), guidelines for treatment (GPs feel compelled to prescribe by the present guidelines) and organization of healthcare (collaboration with prescribing medical specialists and dispensing pharmacists.

**Conclusions:**

The GPs’ beliefs concerning elderly patients are a barrier to explore patient preferences when reviewing preventive medication. GPs would welcome decision support when dealing with several guidelines for one patient. Explicit rules for collaborating with medical specialists in this field are required. Training in shared decision making could help GPs to elicit patient preferences.

## Background

Elderly patients with multimorbidity who receive treatment according to professional guidelines for their respective diseases use a large number of drugs. Preventive medication, such as for cardiovascular risk management and the treatment of diabetes mellitus, contributes substantially to this number [1]. Clear information on the effects of these combined therapies in elderly patients is often lacking [2]. The respective risk reductions found in clinical trials are probably not strictly cumulative, and the benefits to these elderly patients will be less than the sum of the ‘single’ therapies [3]. The number of drugs, however, may burden patients and enhance the risk of adverse drug events (ADEs). Polypharmacy is associated with increased non-adherence to prescribed medications, as well as with diverse outcomes such as malnutrition, functional impairment, falls and fractures, hospitalization and institutionalization [4,5].

Patients and doctors may be inclined to accept ADEs as unavoidable [6] but elderly patients can also have unvoiced concerns about the need to take all their medication [7]. They value some drugs as being more important than others [8] but also differ in their preferences for intensive treatment [9]. Their health goals focus more on quality of life than on extending their lives [10]. In view of the limited life expectancy of many of these older patients, stopping medication meant for prevention may relieve symptoms perceived as ADEs, and thus improve the patient’s wellbeing. To support doctors in their management of polypharmacy in the elderly, two kinds of expert-driven instruments have been developed. Firstly, there are several protocols for reviewing medication using criteria for drug and patient characteristics, such as comorbidity and pharmacodynamics [11]. Secondly, a normative model has been developed for good prescription practices for elderly patients [12]

Thus far, these medication-centred tools, which have been developed from the expert’s point of view, have demonstrated little effect on patient outcomes, in particular on mortality or quality of life [13]. As this latter goal is the most important for patients late in life, physicians should focus on patient preferences to improve quality of life [14]. This is also emphasized by Holmes who points out how treatment targets and goals of care for the individual patient should be adapted over time according to the preferences of the patient and his life expectancy [12].

In recent years, much attention has been paid to shared decision-making in general practice, mainly focusing on patient preferences [15]. In diabetes care it has been found that patients and their doctors generally differ in treatment goals [16], but no attention has been paid to how such issues affect decisions to stop treatment for the very elderly.

This study explores how experienced GPs feel about deprescribing medication in older patients with multimorbidity and to what extent they involve patients in these decisions.

## Methods

We chose a qualitative study design as data about deprescribing medication in elderly patients by GPs are lacking sofar. A small group meeting of peers seemed the best setting to create a non-judging atmosphere in which the GPs would stimulate and inspire each other to exchange their views and experiences with this issue. A focus group can provide such a climate. On basis of these conversations we intended to develop a conceptual framework for understanding and categorizing GPs’ views on the subject.

We organized focus groups of GPs with a minimum of five years experience and active as GP trainers. We thus ensured that our participants had sufficient exposure to the relevant problems and as GP trainers were used to reflecting on their daily practice routines.

GP trainers with a third-year trainee in their practice at the time of the study were invited by the department of vocational training for general practice to participate in the study. Recruitment of participants continued until saturation was reached.

In December 2010 and January 2011 three focus groups were organized in which 12, 9 and 8 GPs participated, respectively. By the third focus group, most of the issues that were being raised had already been mentioned, and it was decided that little was to be gained from continuing data collection, in other words that saturation was reached.

Having read the study protocol the secretary of the medical ethical board of the University Medical Center Groningen gave the advice not to seek a formal ethical approval of the committee on grounds of efficiency, as the participating GPs were asked to give their explicit consent to the anonymous publication of the data at the end of a meeting.

Before starting, participants completed a brief questionnaire on demographic and practice characteristics. The groups were led by a moderator (HJG) with one observer (JS). At the start of the meeting the focus group moderator presented a hypothetical profile of a very elderly patient with multimorbidity and her guideline-driven list of medication (Table 1). This example was used to help participants to remember their own similar patients. The moderator outlined the position of the GP balancing between the risks of polypharmacy, the vulnerability of elderly patients and the guidelines involved recommending prescribing. Participants were encouraged to share their views and experiences on the possibilities for reducing the medication of their patients.

**Table 1:** Fictitious profile of a very elderly patient with multimorbidity

·	Alendronate tab 70 mg q1w
·	Calciumcarb/colecalcif 500 mg/400IE BID
·	Acetylsalicyl tab 80 mg OD
·	Dipyridamol caps mga 200 mg BID
·	Enalapril tab 10 mg OD
·	Gliclazide tab mga 30 mg OD
·	HydroChloroThiazide tab 12.5 mg OD
·	Metformin tab 500 mg TID
·	Metoprolol tab mga 50 mg OD
·	Macrogol 2dd1 BID
·	Paracetamol tab 500 mg TID
·	Ranitidine tab 150 mg BID
·	Salmeterol inhal 50 mcg BID
·	Simvastatine tab 40 mg OD
·	Spironolacton tab 25 mg OD 0.5
·	Tiotropium inhal 18 mcg OD
·	Temazepam tab 10 mg OD

When necessary, question probes were used to elicit discussion on key topic areas such as the elements of the Holmes model (life expectancy, treatment targets, goals of care and time until benefit) that did not emerge spontaneously in the conversation.

### Data analysis and intersubjectivity

The focus group meetings were held at the Department of General Practice of the University Medical Center Groningen. The meetings lasted about 2 hours and were audiotaped. The tapes were transcribed verbatim. These manuscripts were read by the first and second author, who independently selected relevant text fragments. They then labelled these fragments and sorted them. Central themes were extracted from these labelled and sorted fragments. When the two researchers could not agree, the issue would be presented to the third author (LJGV), whose view was final. The stepwise procedure of selection, labelling and extraction was documented.

## Results

### Characteristics of participants

Of the participants, two were female and the mean age was 54 (range 39–65); 13 practices were located in a city, 9 practices in the countryside and 7 practices in between. All participants were practising in the northern three provinces of the Netherlands.

During the process of analysing the transcripts and labelling fragments non-agreement between the two researchers did not occur. The following themes were grouped into three domains:

1. Beliefs of the GPs concerning, best practice, patients and medication

2. Knowledge and skills of the GPs

3. Guidelines for treatment and organization of healthcare

### Beliefs of the GPs

In general, GPs report to support the concept of a patient-centred management as best practice. Respect for values and beliefs of the patient are a compass for their management. Discussing drug therapy with their patients, they see it as their duty to provide necessary information on possible treatment choices and their respective outcomes.

Above all you should provide sufficient information to enable people to make a choice. Honest and good information.

Let people choose for themselves however. Not based on emotions but numbers. Explain it with percentages.

In practice, however, they experience a number of problems with actually addressing the issue of deprescribing medication. Based on their experience, they consider that their patients have no problem with polypharmacy, or with medication burden,

The discontent rarely lies with old people themselves.

but on the other hand they are aware of the importance to know how a patient values his medication

I think that it’s important how people themselves look onto their medication.

Some GPs indicate that patients appear to cling to their extensive medicationlist.

..some patients love all those drug, to show off to their neighbours.’ You know what mass I have to take each day?’

Nonetheless, the GPs acknowledge that they may not be fully aware of the actual problems patients experience. In the GPs’ view, patients underreport possible ADEs, attributing these symptoms to old age rather than to their medication.

They accept these symptoms, they are part of their aging.

In addition, patients may report their symptoms to other healthcare professionals, for example, to the medical specialist, nurse practitioners or specialist nurses. Moreover, GPs are reluctant to initiate a discussion about stopping medication because they are concerned that patients may interpret this as a sign of being given up on.

People may then get the feeling, ‘Don’t I count anymore, am I not important?’

The GPs hesitate to discuss the subject of life expectancy with their patients.

I think it’s tricky to discuss life expectancy with a patient.

On the other hand some participants report how patients spontaneously talk about their limited life expectancy.

‘What ‘s left for me, is limited.’

‘Well doc, when it’s over tomorrow, that’s OK with me’.

Some GPs think that confronting a patient with a discussion about life expectancy versus quality of life is not ethical. At the same time, however, others report that a discussion with their patient about the quality of the patient’s remaining lifetime had a positive effect on their relationship.

Participants vary in their belief on the effects of preventive drug therapy in elderly patients. Some state that the benefit of preventive medication for the individual patient is small, but at a population level worthwile.

I think these (ARR) numbers are low; these numbers are disappointing.

Finally, some GPs mention patient characteristics as a barrier to the patient’s understanding of the issue.

Low education and old age means it is more difficult to discuss these matters.

### Knowledge and skills

Confronted with multimorbidity and its ensuing problems GPs experience difficulties in identifying ADEs, and take the patient’s judgement in this matter seriously.

Which drugs do you think are responsible? Patients are mostly right.

During the meetings it became clear that there is an obvious difference for GPs between stopping medication on account of symptoms and intervening in long-term preventive medication. Participants felt competent in symptom management, stopping medication when symptoms are cured or relieved. For chronic problems, the symptomatic medication was tailored to an optimum as indicated by the patient’s feedback. Preventive medication, however, did not offer such a compass. The real area of concern for the participants was how to manage the long-term use of preventive medication.

In my view there’s a big difference between drugs meant for prevention and the drugs that are really therapeutic.

I focus first on preventive medication: which drug is really indicated?

The problem of the lack of evidence of the effects of preventive medication in the very elderly is paramount

With a 40 year old, I’m fairly confident deciding on what medication to prescribe. But I notice I’m less certain with an 80 year old.

I take her quality of life into account and ask myself will she live long enough to benefit from this (preventive) drug?

GPs indicate a strong need for clear information on the benefit/risk ratio of preventive medication in the very old and often frail.

Giving the patient real choices, providing information is very useful. For instance, by putting ARR numbers on the package.

Even if such information were available, some participants feel incompetent in risk communication, and others consider this information not helpful for actual shared decision-making.

We can’t predict the effect for the individual patient.

The coloured numbers in the cardiovascular risk tables of our guideline have an important effect: when your patient sees himself land in orange or red his motivation is influenced.

All participants admit they were seldom aware of their patients’ treatment preferences.

Where some participants express problems with explaining the tension between quality and length of life,

An elderly person stands up, feels dizzy as hell, but he may live a little longer. Well, these matters I discuss.

others emphasize the option of taking a positive approach to stopping preventive medication.

You can have a field day with crossing off medication: ‘sure, scrap half of it’.

### Guidelines and organization of healthcare

Another important group of barriers concerns the current guidelines. GPs feel forced by current guidelines to prescribe many different medicines: they appear to pile the recommendations of one guideline on another instead of prioritizing.

To me, the guidelines are kind of a hindrance. At the moment they do not cater for older patients.

Participants claim they often feel guilty when their adherence to guidelines is not up to scratch.

I have difficulty not following the guidelines if I don’t have good reasons to do so.

A new patient entering the practice list is welcomed as an opportunity to review their medication. Some GPs complain about an inadequate overview of the patient’s medication.

The problem is that the medication lists of the doctors involved are not exchanged and are consequently inconsistent.

In multimorbidity, several healthcare providers are involved in a patient’s treatment and communication is sometimes poor. Cooperation with prescribing medical specialists who represent ‘their’ guideline is the next important barrier to deprescribing medication.

All doctors should speak with one voice. Different stories provoke distrust.

In addition, most GPs work closely with a local pharmacist: the task perception of such pharmacists was an important factor when a GP was looking for decision support in medication review.

## Discussion

The participants expressed great commitment to the problems discussed and qualified this issue as highly relevant for general practice. Although they were enthusiastic to contribute to the discussion, their feelings with regard to their management of the problem ranged from moderate optimism to something close to despair. The latter was illustrated by GPs expressing strong concerns about the wellbeing of their elderly patients, but feeling unable to do anything about the medication.

### Summary of main findings

This study reveals a range of factors affecting the GPs’ deprescribing for elderly patients with multimorbidity.The GPs discern symptomatic and preventive medication; deprescribing the latter category is seen as more difficult by the GPs due to lack of benefit/risk information for these patients.

Our findings suggest that GPs tend to avoid discussing withdrawal of preventive medication with their elderly patients. Their beliefs concerning patients support, even justify, this policy. Although the GPs are in favour of a patient-centred approach, they do not inquire into the patients’ preferences or discuss treatment goals.

The GPs indicate a strong need for better information on the benefit/risk ratio for preventive medication in the very elderly. Current guidelines are overly focused on the management of a single disease and do not take into account the problem of multimorbidity. The problem is aggravated by the involvement of several medical specialists, who each emphasize the importance of ‘their’ guideline. Prioritization is clearly needed but GPs do not feel empowered to do so. It is however doubtful that managing polypharmacy of elderly patients with multimorbidity can be completely ‘covered’ by guidelines in view of the heterogeneity of patient characteristics. This underlines the necessity of patient involvement as described in the model of Holmes. The concept of shared decision making provides an important and useful framework to put this model into practice [17].

The GPs’ beliefs about best-practice support management favouring shared decision-making. Apparently there is a discrepancy between this belief in best practice and the actual management of the problem.

### Strengths and weaknesses

Our respondents are not a representative sample of Dutch GPs but a rather homogeneous group of older male GPs. We purposively sampled experienced GPs to ensure sufficient exposure to the theme of this study. The GPs in these focus groups showed a strong involvement with the problem although there was no agreement on the weight attached to the factors that were brought up. In view of the growing population of older patients we don’t expect less involvement among Dutch GPs in general. In our opinion younger GPs tend to follow the guidelines more strictly. Younger GPs whose working alliance with their patient has a shorter history may have more difficulty in anticipating preferences of the patient.

### Comparison with existing literature

Our participants did not bring up non-adherence to complex treatment regimes as a factor influencing their management, but they did struggle with the uncertainties of applying disease specific guidelines to their elderly patients as described by Fried [18]. Their need for better outcome data and the complicating role of prescribing subspecialists was evident.

Aiming for optimal quality of life for the very elderly GPs need the tools of shared decision-making to achieve this goal [17]. Although this emphasizes the importance of exploring patient preferences about treatment goals, in practice GPs appear hesitant. Such reluctance could be overcome by tailoring the training of care providers in communication competences [19]. In particular, GPs need to overcome barriers to discussing life expectancy issues and the length of time to see benefits when considering the pros and cons of preventive medication. Allthough some studies have shown that more than half of the patients are willing to discuss life expectancy [20-22], others report that many seriously ill patients and caregivers may not be ready or able to receive prognostic information [23]. However the number of studies is limited and concerns younger patients in a different setting. Our study focuses on settings that offer an opportunity for discussion on ‘modification of targets for chronic disease management’ and ‘reducing burden of medication’ or ‘life choices’ [21]. Patients as well as caregivers indicated that they would use a shared decision making instrument in clinical encounters and attributed multiple functions to the instrument, especially as a tool to facilitate agreement on treatment goals and plans [24]. Such a simple tool to elicit patient preferences has been developed and looks promising, but questions about its reliability still need to be answered [25].

Providing information to patients about possible harm and benefits appeared to increase the knowledge of patients and to reduce their decisional conflict, but it had no consistent effect on patient’s decision to start or continue drug treatment [26].

Doctors have several explicit reasons for not prescribing according to guidelines in older patients, such as patient preferences, adverse events, non-adherence and patient benefit, but these studies implicitly refer to starting new medication [27]. Intervening in long-term preventive medication is quite another issue, as our results show. The finding that antihypertensive agents can be stopped in elderly patients without harm to them may encourage GPs to seriously consider stopping preventive medication [28].

## Conclusions

Our study is a first exploration of GP perceptions of deprescribing. More generalizable information is required to develop tools that could support GPs when reviewing medication of elderly patients with multimorbidity.

The GPs appear to adhere to multiple guidelines in the absence of a practical tool for prioritization according to patient preferences elderly patients are quite capable of prioritizing outcomes [29]. Such prioritization is essential, as the available expert systems are not sufficient to achieve improvement in quality of life of the elderly patients. The variability in individual priorities underlines the need to elicit individual preferences in a context of shared decision-making. Our results confirm previous findings concerning the need for clear and accessible data on the benefits and risks of treatment options caregivers experience. Such information is a precondition for shared decision-making.

### Implications for future research

The results of our study need to be quantified in a more representative sample of GPs and other healthcare professionals actively involved in treating the frail elderly. Confirmation of our findings will contribute to the development of strategies to overcome the barriers found in this study, with a focus on the training of GPs and their staff. Such project should include the following steps:

1. Training to elicit patient preferences with a tool as described by Fried as a start of medication reviewing. Examples of how a care provider can discuss the option of withdrawal of preventive medication with their patients can be shown and will help to overcome the barriers described above.

2. Training how to translate these patient preferences in congruent changes of the patient’s current treatment.

3. Monitoring the effects of this patient driven medication review on the patient’s wellbeing.

## Competing interests

There are no competing interests.

## Authors contributions

JS and HJG developed the study project, observed the focusgroup meetings and analysed the verbatims. Where no agreement was reached on the labelling of text fragments LJGV made the final decision. FMHR advised about the study design. All authors contributed to the writing of the manuscript and approved the final version.

## Pre-publication history

The pre-publication history for this paper can be accessed here:

http://www.biomedcentral.com/1471-2296/13/56/prepub
